# The PARP1 Inhibitor Niraparib Represses DNA Damage Repair and Synergizes with Temozolomide for Antimyeloma Effects

**DOI:** 10.1155/2022/2800488

**Published:** 2022-04-05

**Authors:** Hong-Yuan Shen, Hai-Long Tang, Yan-Hua Zheng, Juan Feng, Bao-Xia Dong, Xie-Qun Chen

**Affiliations:** ^1^Department of Hematology, Xijing Hospital, Air Force Medical University, Xi'an, 710032 Shaanxi, China; ^2^Institute of Hematology, Northwest University, Xi'an, 710069 Shaanxi, China; ^3^Department of Hematology, Affiliated Hospital, Northwest University, Xi'an, 710082 Shaanxi, China

## Abstract

**Purpose:**

Poly(ADP-ribose) polymerase 1 (PARP1) is necessary for single-strand break (SSB) repair by sensing DNA breaks and facilitating DNA repair through poly ADP-ribosylation of several DNA-binding and repair proteins. Inhibition of PARP1 results in collapsed DNA replication fork and double-strand breaks (DSBs). Accumulation of DSBs goes beyond the capacity of DNA repair response, ultimately resulting in cell death. This work is aimed at assessing the synergistic effects of the DNA-damaging agent temozolomide (TMZ) and the PARP inhibitor niraparib (Nira) in human multiple myeloma (MM) cells.

**Materials and Methods:**

MM RPMI8226 and NCI-H929 cells were administered TMZ and/or Nira for 48 hours. CCK-8 was utilized for cell viability assessment. Cell proliferation and apoptosis were detected flow-cytometrically. Immunofluorescence was performed for detecting *γ*H2A.X expression. Soft-agar colony formation assay was applied to evaluate the antiproliferative effect. The amounts of related proteins were obtained by immunoblot. The combination index was calculated with the CompuSyn software. A human plasmacytoma xenograft model was established to assess the anti-MM effects *in vivo*. The anti-MM activities of TMZ and/or Nira were evaluated by H&E staining, IHC, and the TUNEL assay.

**Results:**

The results demonstrated that cotreatment with TMZ and Nira promoted DNA damage, cell cycle arrest, and apoptotic death in cultured cells but also reduced MM xenograft growth in nude mice, yielding highly synergistic effects. Immunoblot revealed that TMZ and Nira cotreatment markedly increased the expression of p-ATM, p-CHK2, RAD51, and *γ*H2A.X, indicating the suppression of DNA damage response (DDR) and elevated DSB accumulation.

**Conclusion:**

Inhibition of PARP1 sensitizes genotoxic agents and represents an important therapeutic approach for MM. These findings provide preliminary evidence for combining PARP1 inhibitors with TMZ for MM treatment.

## 1. Introduction

Multiple myeloma (MM) represents a hematologic cancer caused by clonal plasma cell growth in the bone marrow [1]. Combination of proteasome inhibitor and immunomodulatory drug and myeloablative high-dose treatment plus autologous stem cell transplantation (ASCT) is efficient therapeutic approach for MM [2, 3]. However, almost all MM patients eventually develop refractory disease and relapse. To date, MM remains an incurable disease, and new treatment approaches are urgently required for improving patient outcome. MM features common chromosomal instability and deranged DNA repair [4, 5]. Cells can initiate multiple DNA repair mechanisms to cope with genotoxic stress such as nucleobase adduct removal and single- (SSB) and/or double-strand DNA break (DSB) repair. Suppressing DNA repair is considered a reasonable sensitization strategy to improve genotoxic therapy [6].

PARP1 represents an important component of the base excision repair (BER) of SSBs [6]. PARP1 suppression promotes SSB accumulation and PARP1-DNA interactions. Insufficient SSB repair results in DSBs during DNA replication, and PARP1 trapping inhibits replication fork generation [7]. Inhibition of PARP1 alters DNA repair, indicating PARP1 suppressors could enhance the cytotoxicity of drugs. Mounting evidence suggests PARP1 suppressors enhance the antitumor properties of alkylating agents such as cisplatin, oxaliplatin, cyclophosphamide, and temozolomide [8–10]. Temozolomide (TMZ) is an alkylating product employed in glioma and leukemia treatment. TMZ damages the DNA via methyl adduct addition to N^7^ guanine (70% of all adducts), N^3^ adenine (9%), and O^6^ guanine (5%) [11]. TMZ causes SSBs, cell cycle arrest, and apoptotic death [12]. However, the doses applied for TMZ monotherapy are usually high enough to cause intolerable toxicity to normal cells. Thus, a combination strategy based on synergistic effects may be a better approach to counter tumor progression and reduce toxicity, ultimately improving disease prognosis. PARP inhibitors have been examined in multiple tumors, e.g., small-cell lung cancer, non-small-cell lung cancer, lymphoma, pancreatic cancer, ovarian cancer, breast cancer, prostate cancer, and Ewing's sarcoma, and improve TMZ's anticancer activities *in vitro* and in xenograft models [13–16]. However, in the field of MM therapy, combination of TMZ and PARP inhibitors has not been previously reported.

Here, TMZ and the PARP inhibitor niraparib were examined for synergism. We hypothesized that TMZ could cause SSBs in MM cells, resulting in SSB buildup and DNA replication fork collapse as well as the generation of lethal DSBs in combination with PARP1 inhibitors. The results provide evidence PARP inhibition has little effects when used as a single agent on MM cells but could remarkably enhance TMZ cytotoxicity both in cultured cells and in mice.

## 2. Materials and Methods

### 2.1. Cell Lines

Human MM RPMI8226 and NCI-H929 cells, provided by the American Type Culture Collection (ATCC), underwent culture in RPMI 1640 (Gibco) containing 10% fetal bovine serum (FBS; Sigma) and 1% penicillin/streptomycin (Hyclone) at 37°C in a humid atmosphere with 5% CO_2_.

### 2.2. Drugs and Chemicals

TMZ (MedChemExpress), Nira (MK4827; MedChemExpress), and niraparib hydrochloride (MK4827 hydrochloride; MedChemExpress) were maintained dissolved in dimethyl sulfoxide (DMSO). DMSO level was always below 5% in all treatments. Antibodies against-gamma H2A.X (phospho S139) (*γ*H2A.X) (ab81299), ATM (ab32420), ATM (phospho S1981, ab81292), RAD51 (ab133534), cyclin D1 (ab134175), CHK2 (ab109413), CHK2 (phospho T68, ab32148), and GAPDH (ab128915) were obtained from Abcam; antibodies targeting cleaved caspase-3 (9664S) and anti-rabbit HRP secondary antibodies (7074) were provided by Cell Signaling Technology.

### 2.3. Cell Viability Assay

A total of 2 × 10^4^ indicated MM cells underwent seeding into a 96-well plate and culture for 4 h, followed by the administration of different doses of TMZ and/or Nira for 48 h. After drug exposure for a specific time, cell viability was examined with Cell Counting Kit-8 (CCK8, China). After exposure for 48 h with the drugs, adding 20 *μ*L of CCK-8 solution to each well, the absorbance at 450 nm with a microplate reader was recorded after incubation for 2 h. CCK-8 kit uses a water-soluble tetrazolium salt to quantify the number of live cells by producing an orange formazan dye. The amount of formazan produced is directly proportional to the number of living cells.

### 2.4. Cell Apoptosis Analysis

RPMI8226 and NCI-H929 underwent seeding in a 6-well plate at 2.0 × 10^5^/well. Cells were treated for 48 h with DMSO, 30 *μ*M TMZ (RPMI8226), 20 *μ*M TMZ (NCI-H929), and 3 *μ*M Nira, respectively, for both cell lines, or combined 3 *μ*M Nira and 30 *μ*M (RPMI8226) or 20 *μ*M (NCI-H929) TMZ for 48 h. The Annexin V/propidium iodide (PI) detection kit (BD Pharmingen™) was utilized for apoptosis quantitation. In brief, after treatment with specific drugs for 48 h, the cells underwent incubation, shielded from light at ambient, with Annexin V/FITC and PI for 15 min. Analysis was performed flow-cytometrically with an Epics flow cytometer. After treatment for 48 h with the drugs, adding 5 *μ*L Annexin V and 15 *μ*L PI for each sample, and incubation in the dark for 15 min, apoptosis analysis was performed by flow cytometer. Cells that were Annexin V/FITC positive (with translocation of membrane phospholipid phosphatidylserine (ps) from the inner to the outer leaflet of the plasma membrane) and PI negative (with intact cellular membrane excluding PI) were regarded as early apoptotic cells, whereas positivity for both Annexin V/FITC and PI was considered as late apoptotic or necrotic cells.

### 2.5. EdU Assay

An EdU Staining Proliferation Kit (iFluor 647) (Abcam, ab222421) was utilized in these assays. After drug treatment, the culture medium was supplemented with 20 *μ*M EdU staining solution and incubated for 2 h at 37°C. This was followed by 4% formalin fixation. An Epics flow cytometer was utilized for analysis. After exposure for 48 h with the drugs, cells were incubated with 20 *μ*M EdU (Abcam, ab222421) for 2 h, followed by fixation, permeabilization, and EdU staining according to the manufacturer's instructions; the EdU-positive cells were determined using flow cytometer.

### 2.6. Soft-Agar Clonogenic Assay

Actively growing cells underwent counting and resuspension in 0.3% agar in RPMI 1640 (maintained liquid at 41°C) containing 10% FCS and specific drugs. This was followed by plating on 0.5% agar in a 24-well plate (1 × 10^4^/well) and incubation under standard conditions for 14–21 days. Colonies in each well underwent 0.5% crystal violet staining. Colony counting was performed under an inverted microscope (Leica, Germany).

### 2.7. Immunofluorescence

RPMI8226 and NCI-H929 cells were incubated with specific drugs at 37°C for 48 h in four groups (DMSO, TMZ, Nira, and TMZ plus Nira). After treatment, cells were harvested, washed, and dropped on adhesive slides. This was followed by fixation with 4% formalin for 15 min and permeabilization and blocking using PBS with 0.4% Triton X-100 and 2% BSA in PBS, respectively. Next, successive incubations with rabbit monoclonal anti-*γ*H2A.X antibodies (Abcam, 1 : 200) and Cy3-linked goat anti-rabbit secondary antibodies were followed by DAPI counterstaining. Cells with >10 nuclear foci were assessed for percentage, among at least 100 cells counted in total.

### 2.8. Immunoblot

After treatment, the RIPA buffer containing protease inhibitors (Thermo Fisher) was utilized for cell lysis. A BCA assay kit (Beyotime) was used for protein quantitation. Proteins underwent separation by 10-12% SDS-PAGE and electrotransfer onto PVDF (polyvinylidene difluoride) membranes (Millipore), which were blocked with 5% skimmed milk in 1× TBST. This was followed by successive incubations with primary and HRP-linked secondary antibodies (CST). SuperSignal reagent (Millipore) was used for visualizing immunoreactive bands.

### 2.9. In Vivo Xenograft Mouse Model

All procedures were carried out following the Guide for the Care and Use of Laboratory Animals by the US National Institutes of Health. The study had approval from the Animal Ethics Committee of Xijing Hospital, Air Force Military Medical University. Briefly, 4–6-week-old female BALB/c nude mice (16–20 g, Charles River Laboratories) underwent subcutaneous inoculation of 1.0 × 10^7^ RPMI8226 cells in 150 *μ*l 50% Matrigel (Corning) in serum-free RPMI 1640. About 7-10 days postcell injection, with tumors measuring about 100 mm^3^, the animals were randomly assigned to 4 groups (each *N* = 5): control group (saline containing 50% PEG300, intraperitoneally (i.p.) administered 5 days/week), TMZ (30 mg/kg injected i.p. 5 days/week), Nira hydrochloride (20 mg/kg administered i.p. 5 days/week), and the TMZ and Nira combination group. A Vernier caliper was utilized to measure the tumors' long- (*a*) and short- (*b*) axis diameters for 21 days at 3-day interval. Tumor volume was derived as *V* = 0.5*a* × *b*^2^. The mouse weight was also recorded by an electronic balance. At study end, euthanasia was carried out with humane methods. The xenografts were histologically analyzed.

### 2.10. Immunohistochemical Staining and TUNEL Assay

Tumor xenograft tissue samples underwent fixation with formalin, paraffin embedding, and sectioning at 5 *μ*m. The sections underwent deparaffinization and rehydration with graded alcohol dilutions for immunohistochemistry. After sequential incubation with primary (overnight at 4°C) and secondary (37°C for 30 min) antibodies, the specimens underwent treatment with streptavidin-HRP. The DAB kit was utilized for visualization. Anti-Ki67 (Abcam, 1 : 200), anti-cleaved caspase-3 (1 : 200, CST), anti-RAD51 (Abcam, 1 : 200), and anti-*γ*H2A.X (Abcam, 1 : 200) primary antibodies were utilized. For histological analysis, specimens were examined after hematoxylin and eosin (H&E) staining to identify morphological changes. Tissue specimens were examined using a light microscope (Zeiss, Germany). Furthermore, detection of in situ apoptosis was carried out by TUNEL assay with the In Situ Cell Death Detection Kit, POD (Roche, USA) as directed by the manufacturer.

### 2.11. Drug Synergy and Combination Index

The CompuSyn software was utilized for combination index assessment [17], with CI < 1, CI = 1, and CI > 1 indicating synergistic, additive, and antagonistic effects, respectively.

### 2.12. Statistical Analysis

Data analysis utilized GraphPad Prism v8.0 (San Diego, CA). One-way analysis of variance was performed with Statistical Package for Social Sciences (SPSS) v22.0. All assays were performed thrice, and data are mean ± standard deviation (SD).

## 3. Results

### 3.1. Nira Enhances the Toxicity of TMZ in MM Cell Lines

**Figure 1:**
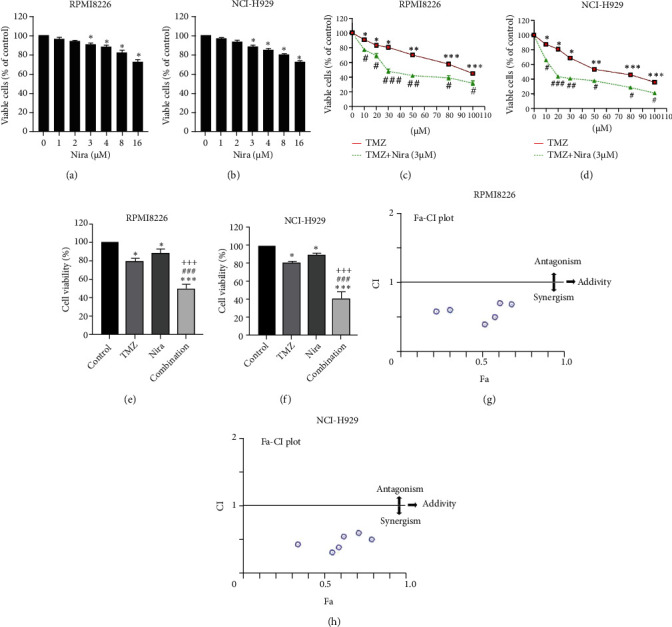
Nira induces synergistic cytotoxicity with TMZ in MM cell lines. (a) RPMI8226 and (b) NCI-H929 cells were exposed to increased Nira amounts for 48 h, before CCK-8 assay analysis of cell viability. (c) D MM cells were incubated for 48 h with increasing doses of TMZ and Nira (3 *μ*M), either alone or in combination, followed by the CCK-8 assay. (e, f) MM cells were administered TMZ and/or Nira for 48 h (RPMI8226 cells, 30 *μ*M and 3 *μ*M, respectively, and NCI-H929 cells, 20 *μ*M and 3 *μ*M, respectively), followed by the CCK-8 assay. (g, h) Fa–CI plots according to the Chou–Talalay equation, generated by CompuSyn v1.0. Round symbol indicates CI (combination index) for a given Fa (fraction affected) at each dose. ^∗^*P* < 0.05, ^∗∗^*P* < 0.01, and ^∗∗∗^*P* < 0.001 versus control group; ^#^*P* < 0.05, ^##^*P* < 0.01, and ^###^*P* < 0.001 versus TMZ group; ^+++^*P* < 0.001 versus Nira group. TMZ: temozolomide; Nira: niraparib.

For treatment of ovarian cancer, the recommended dose of niraparib is 300 mg per day for 21 days every 28-day cycle, with plasma C max approximating 2 *μ*M following the initial treatment and rising to 3.5~4.2 *μ*M at day 21 [18]. We first tested whether the PARP inhibitor Nira monotherapy could elicit direct cytotoxicity on MM cells *in vitro*. At physiological concentrations (≤3.5 *μ*M), Nira caused no significant cytotoxicity in RPMI8226 and NCI-H929 cells, as depicted in Figures 1(a) and 1(b). Next, the effects of fixed low-dose concentrations of Nira combined with TMZ on the viability of MM cells were examined with CCK-8. Upon 48 h of incubation, TMZ monotherapy markedly suppressed viability in MM cells in comparison with control cells, concentration-dependently (Figures 1(c) and 1(d)). We used the physiological concentration of Nira at 3 *μ*M for subsequent experiments. When cells were cotreated with a fixed dose of Nira and different doses of TMZ, as depicted in Figures 1(c) and 1(d), the IC_50_ of TMZ in RPMI8226 cells was significantly reduced from 85 *μ*M to 30 *μ*M; the IC_50_ of TMZ in NCI-H929 cells declined from 65 *μ*M to 20 *μ*M. Combination of Nira and TMZ showed very good therapeutic potential for MM cell lines (Figures 1(e) and 1(f)). The CompuSyn software was used to generate CI and Fa-CI plots for varying concentrations of TMZ with fixed dose of Nira to determine the synergistic effects. Figures 1(g) and 1(h) and Tables 1 and 2 indicated synergistic effects for all the doses tested. Combination of TMZ at 30 *μ*M (RPMI8226 cells) and 20 *μ*M (NCI-H929 cells) with 3 *μ*M Nira showed the strongest synergistic effects. These doses were selected for the next experiments to test whether this combination was optimal.

### 3.2. Effects of TMZ and/or Nira on Apoptosis

**Figure 2:**
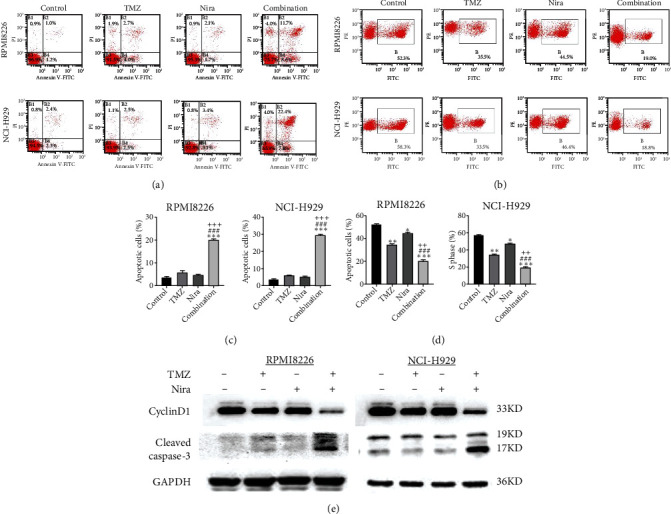
Effects of TMZ and/or Nira on proliferation and apoptotic death in MM cells. (a) RPMI8226 and NCI-H929 cells were administered TMZ and/or Nira for 48 h, and apoptosis was examined flow-cytometrically after Annexin V–FITC/PI staining. (b) RPMI8226 and NCI-H929 cells were administered TMZ and/or Nira for 48 h, and S-phase cells were detected flow-cytometrically by the EdU assay. (c, d) Quantification of apoptotic and S-phase cells shown in (a). (e) Cell cycle-related and apoptosis-associated proteins in RPMI8226 and NCI-H929 cells were quantitated by immunoblot. ^∗^*P* < 0.05, ^∗∗^*P* < 0.01, and ^∗∗∗^*P* < 0.001 versus control group; ^##^*P* < 0.01 and ^###^*P* < 0.001 versus TMZ group; ^++^*P* < 0.01 and ^+++^*P* < 0.001 versus Nira group. TMZ: temozolomide; Nira, niraparib.

In order to assess whether administration of TMZ and/or Nira for 48 h affects apoptosis in MM cells, Annexin V/PI staining of RPMI8226 and NCI-H929 cells was carried out. As presented in Figures 2(a) and 2(c), single-agent TMZ or Nira did not induce significant apoptosis. However, combined treatment with TMZ and Nira induced more apoptosis (25% apoptosis) (*P* < 0.01). We also evaluated the effects of TMZ and/or Nira on apoptosis-associated protein levels in MM cells by immunoblot. Cleaved caspase-3 protein amounts in the combination group were markedly higher compared with controls and TMZ and Nira monotherapies (Figure 2(e)).

### 3.3. Effects of TMZ and/or Nira on Cell Proliferation

**Figure 3:**
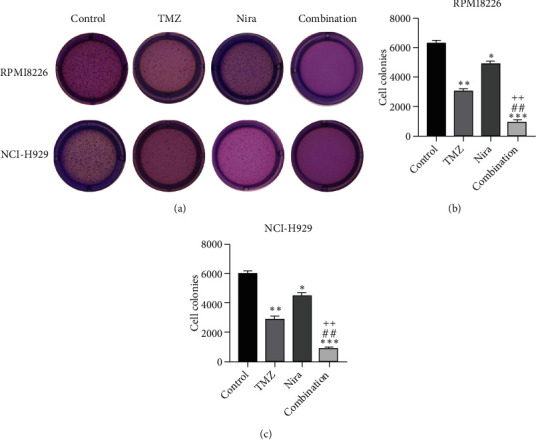
Effects of TMZ and/or Nira on colony formation in MM cells. (a) RPMI8226 and NCI-H929 cells were administered TMZ and/or Nira for 14-21 days, and colony formation ability was determined by soft-agar clonogenic assay. (b, c) Quantification of colonies shown in (a). ^∗^*P* < 0.05, ^∗∗^*P* < 0.01, and ^∗∗∗^*P* < 0.001 versus control group; ^##^*P* < 0.01 versus TMZ group; ^++^*P* < 0.01 versus Nira group. TMZ: temozolomide; Nira: niraparib.

To further examine the above synergistic effects of TMZ and Nira in the present study, this optimal drug combination was assessed for its effects on the S-phase distribution of MM cells treated with TMZ and/or Nira, for 48 h flow-cytometrically. Figures 2(b) and 2(d) show TMZ reduced the proportion of cells in the S-phase at 48 h. Cotreatment with TMZ and Nira significantly enhanced this effect compared with TMZ alone. Furthermore, we performed soft-agar colony formation assay to assess the antiproliferative effect of Nira-TMZ. As depicted in Figures 3(a)–3(c), the amounts of colonies following Nira-TMZ treatment were starkly diminished in comparison with the TMZ monotherapy, Nira monotherapy, and untreated control groups of MM cells. These data suggested that combination of TMZ and Nira markedly inhibited cell proliferation, with potent synergistic cytotoxicity in MM cells. Accordingly, immunoblot showed that the cell cycle-associated protein cyclin D1 was significantly decreased in the combination group compared with the Nira or TMZ alone group (Figure 2(e)).

### 3.4. Combination of Nira and TMZ Induces *γ*H2A.X Foci Formation and Blunts DNA Damage Repair in MM Cell Lines

**Figure 4:**
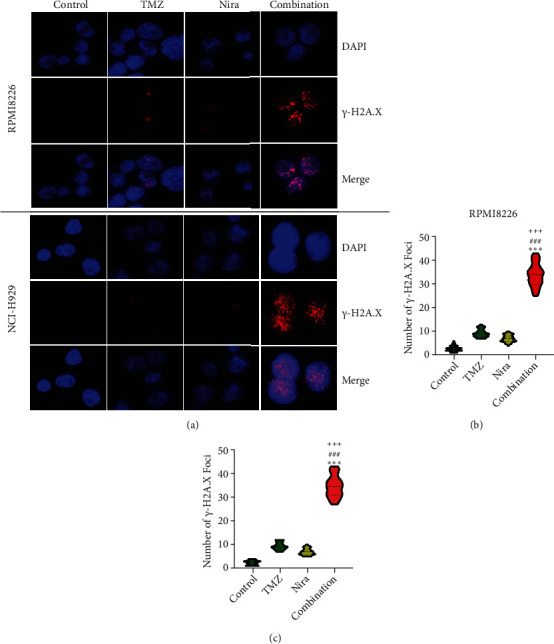
TMZ and/or Nira induce *γ*H2A.X foci formation and block DNA damage repair in MM cell lines. (a) Immunofluorescent staining showing high amounts of *γ*H2A.X foci in MM cells administered the TMZ/Nira combination regimen (TMZ at 30 *μ*M and 20 *μ*M for RPMI8226 and NCI-H929 cells, respectively, and Nira at 3 *μ*M) for 48 h. (b, c) Quantitation of (a). ^∗∗∗^*P* < 0.001 versus control group; ^###^*P* < 0.001 versus TMZ group; ^+++^*P* < 0.001 versus Nira group. TMZ: temozolomide; Nira: niraparib.

We used immunofluorescence to detect whether histone H2A.X phosphorylation (*γ*H2A.X) forms nuclear foci after exposure to TMZ and/or Nira treatment for 48 h. The number of *γ*H2A.X foci is considered to be tightly associated with the amounts of cellular DSBs. The majority cells in the control group had no *γ*H2A.X foci in the nuclei, while TMZ or Nira alone treatment caused sparse *γ*H2A.X foci in MM cells. The proportion of *γ*H2A.X-positive cells and the number of *γ*H2A.X foci per nucleus were overtly increased after Nira plus TMZ treatment (*P* < 0.05) (Figures 4(a)–4(c)), suggesting that the combination induced significant DNA damage. This revealed that the PARP inhibitor Nira hindered DNA damage response (DDR) aroused by TMZ cytotoxicity.

### 3.5. Effects of Nira and/or TMZ on DDR Signaling in MM Cells

**Figure 5:**
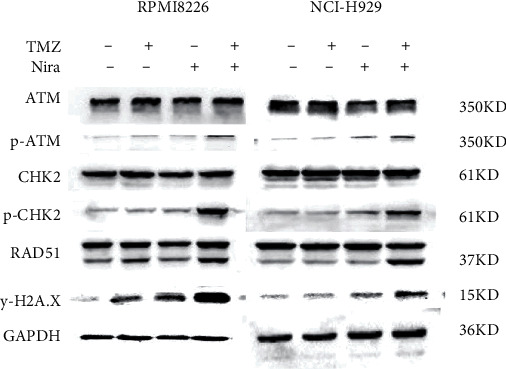
Effects of Nira and/or TMZ on DDR signaling in MM cells. RPMI8226 and NCI-H929 cells were administered TMZ and/or Nira for 48 h at various amounts, and immunoblot was carried out for quantitating proteins. GAPDH was utilized for normalization. TMZ: temozolomide; Nira: niraparib.

In order to elucidate the mechanism responsible for the synergistic effects of Nira and TMZ, the protein levels of DDR signaling effectors were examined. Cotreatment with TMZ plus Nira led to significantly increased p-ATM, p-CHK2, RAD51, and *γ*H2A.X compared with monotherapy (Figure 5), indicating that TMZ-Nira combination therapy could function via the DNA damage response. These proteins are core members of the DDR when DNA confronts genotoxic challenges such as genotoxic chemotherapy. Mounting evidence suggests the BER pathway efficiently removes DNA nucleobase adducts and prevents DNA damage and cell death associated with DNA alkylating agents, including TMZ, cyclophosphamide, and carmustine [19]. Here, the PARP1 inhibitor Nira hampered the recruitment of some core DNA repair proteins of the PARP/BER pathway. Pharmacologically, the PARP1 inhibitor Nira hampered the recruitment of some core DNA repair proteins of the PARP/BER pathway, blocked SSB repair to some extent, and caused SSB accumulation [20, 21]. And thus, unrepaired SSBs were converted into lethal DSBs causing cell death and proliferation arrest in MM cells, which could explain the synergistic effects of TMZ and Nira.

### 3.6. In Vivo Effects of Nira and/or TMZ in an RPMI8226 Xenograft Model

**Figure 6:**
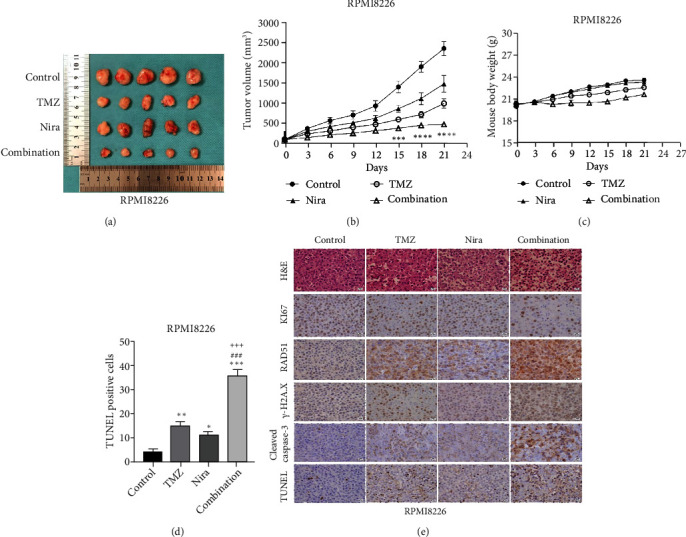
The TMZ/Nira regimen suppresses plasmacytoma xenograft *in vivo*. (a) Human MM cell-derived xenograft model was built using RPMI8226 cells to confirm the synergistic effects of TMZ and Nira. The animals had decreased tumor volumes upon administration of the TMZ-Nira combination compared with the TMZ and Nira monotherapy and control groups. (b, c) Tumor size and weight measurements revealed decreased plasmacytoma xenograft growth after TMZ/Nira cotreatment. (d) The percentages of TUNEL-positive cells were obtained by ImageJ. (e) Apoptotic and proliferating cells in tumor specimens, respectively, were detected by the TUNEL assay, H&E staining, and immunochemistry. The expression levels of *γ*H2A.X, RAD51, Ki67, and cleaved caspase-3 were determined by immunohistochemistry. Reduced expression of Ki67 and elevated expression of *γ*H2A.X, RAD51, and cleaved caspase-3 were found in the TMZ plus Nira group. Mean ± SD (*n* = 5 per group). Significant differences were indicated as follows: ^∗^*P* < 0.05, ^∗∗^*P* < 0.01, ^∗∗∗^*P* < 0.001, and ^∗∗∗∗^*P* < 0.0001 versus control group; ^###^*P* < 0.001 versus TMZ group; ^+++^*P* < 0.001 versus Nira group. Scale bar, 20 *μ*m. Original magnification, ×400. TMZ: temozolomide; Nira: niraparib.

The effects of daily TMZ (35 mg/kg) and/or Nira (20 mg/kg), administered i.p. 5 days per week, on RPMI8226 cell growth were examined in a human plasmacytoma xenograft model via subcutaneous injection. TMZ plus Nira regimen resulted in significantly reduced tumor volume and weight over time, compared with the vehicle and single-agent treatment groups (Figures 6(a)–6(c)). During the three weeks of treatment, the combination regimen showed no significant weight loss, and the animals showed good general health and activity, with no signs of discomfort, which showed a good tolerability for the Nira and TMZ combination.

At the end of drug administration, euthanasia was performed, and tumors were extracted and analyzed by immunohistochemistry (IHC) for Ki67 and cleaved caspase-3, which are proliferative and apoptotic biomarkers in tumors. The tumors were also examined by TUNEL assay to assess in situ apoptosis to further confirm the above findings. Apoptotic (TUNEL- and cleaved caspase-3-positive) cells in the combination group were extensively increased as well as the morphologic features of apoptosis (Figures 6(d) and 6(e)). On the contrary, proliferative (Ki67-positive) cells were overtly decreased (Figure 6(e)), in accordance with the previously observed proliferation arrest. Moreover, we demonstrated that *γ*H2A.X and RAD51 expression levels were starkly higher in the combination treatment group (Figure 6(e)), which suggested that the combination regimen enhanced DNA damage and blocked DNA repair. Jointly, these *in vivo* findings about tumor proliferation corroborated those obtained in cultured cells, further verifying our hypothesis that TMZ-Nira cotreatment produces excellent synergistic effects.

## 4. Discussion

MM comprises ~10% of all hematologic cancer cases. MM cases show good response to alkylating agents initially, but the quasi-totality of patients relapse eventually, including those who achieved complete remission (CR) [22]. In addition, many patients develop refractory disease because of multidrug resistance (MDR). Recently, although novel therapeutic approaches (newer proteasome inhibitors, IMiDs, CD38 monoclonal antibody, CAR-T therapy, and autologous stem cell transplantation (ASCT)) have extended survival, many patients still inevitably relapse and die of comorbidities [23]. Many patients are ineligible for auto-ASCT due to advanced age at diagnosis. It is imperative to identify more effective therapeutic options to improve curative effects in elderly and advanced-stage patients [24]. Genotoxic agent-based chemotherapeutic regimens are important in MM treatment. MDR represents the major obstacle hindering prognosis improvement in MM cases. Many factors contribute to MRD such as elevated drug efflux, altered drug resistance-related genes, increased DNA damage repair, and reduced apoptosis [25, 26]. Previous reports have suggested that PARP1 inhibitors synergize with several conventionally applied chemotherapeutics such as TMZ. As shown above, in combination with the PARP1 inhibitor Nira, the IC_50_ of TMZ was reduced significantly. An early event following DNA DSBs is the generation of phosphorylated histone H2AX (*γ*H2A.X), which is considered the gold standard for DSB detection [27]. Therefore, *γ*H2A.X amounts reflect DNA damage resulting from chemotherapeutic agents in cancer cells, representing an index of cell sensitivity to chemotherapy [28]. As shown above, after exposure to combination treatment, upregulation of *γ*H2A.X indicated increased DSBs and enhanced drug sensitivity. RAD51 represents the most important protein that promotes strand pairing and exchange between homologous DNAs during homologous recombination repair (HRR) [29]. In this study, immunoblot and IHC analysis demonstrated elevated expression of RAD51 in the combination group, suggesting severe and lethal DNA DSB accumulation. Tumor cell sensitivity to chemotherapeutics promoting DNA damage is function of the balance between DNA damage and repair. Consequently, targeting key factors in DNA repair response that protect cells from death represent a promising approach for enhancing the curative effect of routine cytotoxic molecules.

PARP1 mainly contributes to SSB repair, particularly via the BER pathway. A PARP1 inhibitor was first successfully used as monotherapy based on the concept of “synthetic lethal therapy” for the treatment of cancers exhibiting intrinsic DNA repair anomalies. BRCA1/2-mutated cancers with abnormal DNA homogenous repair are vulnerable to further DNA repair pathway suppression [30, 31]. The PARP1/BER pathway is critical in clearing chemotherapy-induced DNA adducts, which prevents cell cycle arrest and death. When DNA damage occurs, DNA lesions are recognized by DNA glycosylase that performs hydrolysis of the altered base to produce an apurinic-apyrimidinic (AP) base. This AP base undergoes removal by AP endonuclease for generating a DNA nick, which interacts with PARP1, resulting in DNA polymerase h (Pol h) and DNA ligase complex recruitment for repairing the DNA [32–34]. Temozolomide introduces DNA damage through DNA alkylation or methylation. Under normal conditions, temozolomide promotes methyl adduct formation in DNA at guanine's N7, guanine's O6, and adenine's N3. Since methylpurines (N7-MeG and N3-MeA) undergo repair quickly via BER, cytotoxicity mostly results from methylation at guanine's O6. PARP suppressors affect PARP1 and PARP2, blunt BER, and sensitize malignant cells to temozolomide [35, 36], constituting potential combinatory agents for use with temozolomide in cancer.

This study explored the mechanism by which temozolomide cytotoxicity was potentiated by the PARP suppressor Nira. As demonstrated above, Nira monotherapy yielded about 10% of cell viability inhibition (IC_10_) at the clinical dose. In this study, temozolomide's effect was starkly enhanced by Nira. Indeed, we showed that in MM cells, 48 h of exposure to Nira plus TMZ achieved TMZ potentiation to a large extent. We hypothesized that temozolomide-dependent nucleotide methylation was not effectively repaired with BER blocking by Nira. The produced SSBs were subsequently converted into DSBs, causing MM cell apoptosis and proliferation arrest. Indeed, the amounts of DSBs, reflected by *γ*H2A.X expression, were markedly elevated after combined administration of temozolomide and Nira. The elevated amounts of DSBs are correlated with enhanced cytotoxicity under these conditions. Bryant and Helleday suggested PARP suppression alters endogenous SSB repair, resulting in collapsed DNA replication forks [37]. This study provides some evidence that DSBs are important in temozolomide-dependent cytotoxicity in MM cells. Meanwhile, *γ*H2A.X level was confirmed in the current work as a useful index for assessing the impact of PARP suppression on DNA repair.

PARP suppressors as chemosensitizing agents are scarcely applied in the clinical setting, probably because of the complexities of combination therapies, e.g., identifying the optimal dose [10]. Cotreatment with PARP1 inhibitors and conventional chemotherapeutics was shown to highly enhance the efficacy of chemotherapy that exerts cancer suppressive effects at reduced doses [38].

The combination regimen not only inhibited cancer cell proliferation and induced apoptosis but also reduced human plasmacytoma xenograft growth in mice. Histologic analysis confirmed that suppression of proliferative markers, appearance of severer DNA damage and breaks, and enhanced cell apoptosis corroborated xenograft growth suppression.

A limitation of this study is that the dosing and scheduling of TMZ (30 mg/kg, i.p. ×5, three cycles) used in this animal model were different from the regimen employed in clinical practice. Although the animals in this research did not show signs of discomfort and significant weight loss in the monotherapy and combination groups, the cooperative mechanism of genotoxic agents and PARP1 inhibitors remains to be further elucidated.

## 5. Conclusion

This study confirmed that Nira remarkably enhanced temozolomide's anticancer effects both in cultured cells and in mice. The above preclinical findings provide a sound rationale for the use of Nira for chemosensitization of MM cases to temozolomide in clinic. This research also provides a novel treatment strategy for MM, particularly in patients who have exhausted other treatment modalities. Nira has excellent pharmacokinetic features in many species and could cross the blood-brain barrier [8], making it particularly suitable for combined use with temozolomide for treating intracranial tumors in clinical practice.

## Figures and Tables

**Table 1:** Combination index data for TMZ and Nira in RPMI8226 cells.

Dose TMZ (*μ*M)	Dose Nira (*μ*M)	Combination effect^∗^	CI value
10	3	0.23	0.56554
20	3	0.31	0.58787
30	3	0.52	0.38529
50	3	0.58	0.49207
80	3	0.61	0.6861
100	3	0.68	0.67125

Notes: Combination index (CI) values for TMZ and Nira were based on the Chou–Talalay's method at 48 h. CI < 1, CI = 1, and CI > 1 reflect synergistic, additive, and antagonistic effects, respectively. Independent experiments were performed thrice. ^∗^Mean value of three replicates. In each condition, standard deviation is less than 10%. Abbreviations: TMZ: temozolomide; Nira: niraparib.

**Table 2:** Combination index data for TMZ and Nira in NCI-H929 cells.

Dose TMZ (*μ*M)	Dose Nira (*μ*M)	Combination effect^∗^	CI value
10	3	0.34	0.42312
20	3	0.55	0.3069
30	3	0.59	0.37772
50	3	0.62	0.54191
80	3	0.71	0.58941
100	3	0.79	0.49808

Notes: Combination index (CI) values for TMZ and Nira were based on the Chou–Talalay's method at 48 h. CI < 1, CI = 1, and CI > 1 reflect synergistic, additive, and antagonistic effects, respectively. Independent experiments were performed thrice. ^∗^Mean value of three replicates. In each condition, standard deviation is less than 10%. Abbreviations: TMZ: temozolomide; Nira: niraparib.

## Data Availability

All data throughout the manuscript were generated by our experiments on cells and mice. And data can be obtained by communicating with corresponding author or three co-first authors.
